# Elevated Serum Leptin Levels in Patients With Eosinophilic Chronic Rhinosinusitis

**DOI:** 10.3389/fphar.2021.793607

**Published:** 2022-01-03

**Authors:** Yoshimasa Imoto, Shigeharu Ueki, Yukinori Kato, Kanako Yoshida, Taiyo Morikawa, Yukihiro Kimura, Masanori Kidoguchi, Toshiki Tsutsumiuchi, Keisuke Koyama, Naoto Adachi, Yumi Ito, Kazuhiro Ogi, Masafumi Sakashita, Takechiyo Yamada, Robert P. Schleimer, Tetsuji Takabayashi, Shigeharu Fujieda

**Affiliations:** ^1^ Department of Otorhinolaryngology Head and Neck Surgery, Faculty of Medical Sciences, University of Fukui, Fukui, Japan; ^2^ Department of General Internal Medicine and Clinical Laboratory Medicine, Akita University Graduate School of Medicine, Akita, Japan; ^3^ Department of Otorhinolaryngology, Head and Neck Surgery, Graduate School of Medicine, Akita University, Akita, Japan; ^4^ Division of Allergy and Immunology, Department of Medicine, Northwestern University Feinberg School of Medicine, Chicago, IL, United States

**Keywords:** eosinophilic chronic rhinosinusitis, leptin, eosinophils, eotaxin-3, adipokines

## Abstract

**Background:** Eosinophilic chronic sinusitis (ECRS) is a subtype of CRS with nasal polyps (CRSwNP) that is frequently comorbid with asthma. Notably, ECRS patients often show a high recurrence of NPs after surgical resection. Leptin is a hormone produced by adipocytes that has been implicated in airway inflammatory diseases. However, to date, the role of leptin in ECRS has not been investigated.

**Objective:** To determine whether the serum levels of leptin are altered in patients with ECRS.

**Methods:** In total, 40 patients with ECRS, 15 patients with non-eosinophilic CRS (non-ECRS), and 12 individuals without CRS (control) were included in this study. Patient’s serum leptin levels were assessed, and the number of eosinophils in their NPs were measured through a histological evaluation of the three densest areas with cellular infiltrate beneath the epithelial surface. Finally, nasal fibroblast cultures established from NPs were stimulated with varying concentrations of recombinant leptin *in vitro* to determine whether leptin affects eotaxin-3 (Chemokine (C-C motif) ligand 26 :26: CCL26) expression.

**Results:** The serum leptin levels in both the ECRS and non-ECRS groups were significantly higher than those in the control subjects (*p* < 0.0001 vs. ECRS; *p* < 0.05 vs. non-ECRS). Furthermore, ECRS patients displayed significantly elevated serum leptin levels compared to non-ECRS patients (*p* < 0.001), although there was no difference in body mass index between the groups. Notably, serum leptin levels were correlated with the proportion of eosinophils in peripheral blood (r = 0.3575, *p* < 0.01) and the number of eosinophils in NPs (r = 0.5109, *p* < 0.0001). Serum leptin levels were also correlated with eotaxin-3 mRNA expression in NPs (r = 0.5374, *p* < 0.01). Finally, leptin significantly augmented eotaxin-3 expression in nasal fibroblasts established *in vitro* from NPs in a leptin receptor-dependent manner (*p* < 0.05).

**Conclusion:** Leptin levels are elevated in ECRS patients and may both promote and indicate the severity of ECRS as well as systemic type 2-biased inflammatory responses. Combined, these data indicate that circulating leptin may play a significant role in the development of eosinophilic inflammation in NPs.

## Introduction

Chronic rhinosinusitis (CRS) is a heterogeneous disease characterized by local inflammation of the upper airways and paranasal sinuses for a duration of at least 12 weeks. CRS is one of the most common chronic diseases, and it greatly impairs the quality of life of affected patients (Meltzer et al., 2004; Kern et al., 2008; Schleimer et al., 2009; Bhattacharyya, 2011). While some of the complex pathogenic mechanisms of CRS have been described (Hulse, 2016; Schleimer, 2017), the precise mechanism(s) underlying CRS are not well understood. Clinically, CRS patients are frequently divided into two groups based on the presence or absence of nasal polyps (NPs), namely, CRS with NPs (CRSwNP) and CRS without NPs (CRSsNP). Importantly, each type of CRS shows distinct and varied inflammatory patterns and heterogeneity (Tomassen et al., 2016; Tan et al., 2017). In particular, the pathological features of CRSwNP tissues include high eosinophilic infiltration and a type 2 cytokine profile, especially in Western countries (Van Zele et al., 2006). Other inflammatory cells, such as mast cells, basophils, and type 2 innate lymphoid cells (ILC2s), also infiltrate into NPs, and may orchestrate inflammatory responses (Takabayashi et al., 2012; Mahdavinia et al., 2014; Poposki et al., 2017; Stevens et al., 2021). The clinical features of CRSwNP usually include nasal obstruction and olfactory loss, and all forms of CRS are frequently linked to comorbidities, such as asthma, with severe asthma being highly comorbid with CRSwNP (Lin et al., 2011; Lam et al., 2014). In general, topical and oral corticosteroids are the mainstays of therapy for patients with CRSwNP. However, long term administration of oral corticosteroids can cause severe adverse effects; thus, interest in type 2 targeting biologicals is expanding. Notably, sinus surgery is an effective solution for patients who do not respond to medical therapy. Despite these medical and/or surgical therapies, a significant proportion of patients require repeated sinus surgery due to recurrence (Tokunaga et al., 2015). Therefore, in order to improve the management of CRSwNP, the precise mechanisms related to its recurrence must be elucidated.

Historically, NPs found in patients from Asian countries have been reported to be more likely to have neutrophil-dominant inflammation, whereas those found in patients from Europe and the United States are more often characterized by eosinophilic inflammation (Hu et al., 2012; Katotomichelakis et al., 2013; Wang et al., 2014; Tan et al., 2017). Recently, the criteria for eosinophilic chronic rhinosinusitis (ECRS) were defined by the Japanese Epidemiological Survey of Refractory Eosinophilic Chronic Rhinosinusitis (JESREC) study (Tokunaga et al., 2015).This survey examined the proportion of eosinophils in the blood and tissues of 1,716 patients who underwent sinus surgery, and found that blood eosinophils, ethmoid disease, asthma, and aspirin intolerance were all associated with disease recurrence and the need for further surgical intervention. The study also revealed that the prevalence of eosinophilic polyps in Japan is similar to that in Western countries (Tokunaga et al., 2015). Previous clinical studies have suggested that the adoption of a Western lifestyle with a higher consumption of energy and increased obesity may be possible factors driving the increase in prevalence of allergic diseases over the last few decades (Devereux, 2006). Importantly, adipose tissues not only act as a source of energy storage, but they also secrete adipokines, such as leptin, and cytokines (Wellen and Hotamisligil, 2005), which regulate various body functions and can serve as mediators of inflammation. In fact, it has been suggested that obesity may be a risk factor for the development of asthma (Beuther and Sutherland, 2007), and is also closely linked with the severity of asthma (Camargo et al., 1999; Luder et al., 2004; Mosen et al., 2008; Taveras et al., 2008; Taylor et al., 2008; von Mutius et al., 2001), although the results have been inconsistent (Gurkan et al., 2004; Sood et al., 2006; Quek et al., 2010).

Leptin is a 16-kD protein that is a product of the *ob* gene, which is synthesized and secreted mainly by white adipose tissues (Masuzaki et al., 1997; Bado et al., 1998). Leptin plays a key role in the regulation of appetite, metabolism, and body weight (Sahu, 2003; Barrios-Correa et al., 2018). As an adipokine, leptin promotes inflammation, generates reactive oxygen species, induces the activation of natural killer cells, and enhances macrophage activation, phagocytosis, and cytokine release (Carbone et al., 2012). Furthermore, leptin upregulates leukotriene biosynthesis in alveolar macrophages (Mancuso et al., 2004), which may contribute to the pathogenesis of asthma. Notably, previous studies have shown that serum leptin levels are elevated in patients with asthma compared to healthy controls (Gurkan et al., 2004; Sood et al., 2006; Quek et al., 2010).

Therefore, given that the relationship between leptin and ECRS has not been previously evaluated, we aimed to determine whether serum leptin levels are related to the severity of ECRS.

## Materials and Methods

### Patient Recruitment and Clinical Sample Collection

The diagnosis of sinus disease was made based on patient history, clinical examination, nasal endoscopy, and computed tomography (CT) of the sinuses according to the guidelines of the European Position Paper on Rhinosinusitis and NPs (Fokkens et al., 2012). Patients with CRS were recruited before surgery, from the Department of Otorhinolaryngology, Head & Neck Surgery, University of Fukui, Fukui, Japan. Before sample collection, all subjects provided written informed consent. Patients with an established immunodeficiency, coagulation disorder, diagnosis of classic allergic fungal sinusitis, Churg-Strauss syndrome (eosinophilic granulomatosis with polyangiitis), or cystic fibrosis, and those who were pregnant, were excluded from the study. All patients scheduled for surgery had previously failed to respond to adequate trials of conservative medical therapy (prolonged antibiotic regimens, nasal steroid sprays, oral steroids, saline irrigations, and decongestants) administered to control their symptoms. All subjects were prohibited from taking oral steroids for at least 4 weeks prior to surgery. This study was performed in compliance with the Declaration of Helsinki and Good Clinical Practice, with prior approval from the Ethics Committee of the University of Fukui (20120073). Details of the patient characteristics are included in Table 1.

**TABLE 1:** Characteristics of the subjects.

	Control (*n* = 12)	non- ECRS (*n* = 15)	ECRS (*n* = 40)
Sex, male/female	10/2	10/5	27/13
Age, (years, mean ± SD)	45.7 ± 17.8	48.5 ± 12.8	52.4 ± 13.5
Comorbidity of asthma, yes/no	2/10	1/14	15/25^a^
Total IgE (IU/ml, IQR)	148 (45.3–442.0)	271.0 (44.4–554.0)	254.5 (127–545.8)
CRP (mg/dl, IQR)	0.04 (0.02–0.09)	0.1 (0.02–0.4)	0.05 (0.03–0.2)
BMI (IQR)	22.3 (20.2–24.4)	21.5 (20.7–22.7)	23.1 (20.7–25.0)
White blood cell count (IQR)	5200 (4975–6275)	5300 (4150–6150)	5900.0 (5175–6950)
Neutrophils (%, IQR)	45.7 (43.7–57.2)	61.6 (49.2–65.2)	56.1 (46.0–60.8)
Eosinophils (%, IQR)	4.3 (3.2–7.2)	2.9 (1.3–4.5)	4.9^b^ ^,^ ^c^ (4.2–8.3)
Basophils (%, IQR)	0.9 (0.5–1.0)	0.6 (0.4–0.7)	0.9 (0.7–1.3)
Lymphocytes (%, IQR)	33.5 (25.4–42.0)	27.0 (23.1–34.5)	31.3 (26.3–36.8)
Monocytes (%, IQR)	7.2 (6.0–8.0)	7.1 (6.6–8.8)	7.2 (6.0–8.1)
Eosinophil number in nasal polyp (IQR)		15.0 (3.5–23.0)	98.0^d^ (42.0–218.0)
JESREC score (IQR)		7.0 (6.0–8.0)	13.0^d^ (11.0–15.0)

ECRS, Eosinophilic chronic sinusitis; IQR, interquartile range; CRP, C-reactive protein; BMI, body mass index; JESREC, Japanese Epidemiological Survey of Refractory Eosinophilic Chronic Rhinosinusitis.

a *p* < 0.05 vs. non-ECRS.

b *p* < 0.05 vs. Control.

c *p* < 0.001 vs. non-ECRS.

d *p* < 0.0001 vs. non-ECRS.

Patients were prohibited to take food at least 12 h before surgery, and all serum samples were collected just before the surgery. Serum samples were centrifuged at 3000 × g for 10 min and stored at –80°C until use. Total IgE was measured using the ImmunoCAP method (Pharmacia Diagnostics AB, Uppsala, Sweden). To count the proportions of white blood cells, they were collected with EDTA before surgery and cell differentials were performed using an XN-9000 (Sysmex, Hyogo, Japan).

Uncinate tissues (UT) and NP tissues matched with serum samples were obtained from routine functional endoscopic sinus surgery in patients with ECRS or non-ECRS. Specimens from patients without CRS, to be used as controls, were obtained from patients with a sinonasal cyst, inverted papilloma, or nasal septum deviation.

The JESREC score criteria was used for the diagnosis of ECRS, which includes a scoring system that assesses unilateral or bilateral disease, the presence of NPs, ethmoid dominant CT shadows, and the eosinophil ratio in peripheral blood, as previously described (Tokunaga et al., 2015). A total score higher than 11 was regarded as ECRS. The ECRS group was further classified into three subgroups, i.e., mild, moderate, and severe ECRS groups, according to clinical factors A (i.e., >5% eosinophils in peripheral blood, and ethmoid dominant shadow on CT) and B (i.e., comorbid bronchial asthma, intolerance to aspirin and other nonsteroidal anti-inflammatory drugs).

### Enzyme-Linked Immunosorbent Assays

The serum concentrations of leptin were determined using commercially available enzyme-linked immunosorbent assay (ELISA) kits according to the manufacturer’s protocols (Thermo Fisher Scientific, Waltham, MA, United States). The concentrations of eotaxin-3 in tissue homogenates of cultured nasal fibroblasts were determined with a commercially available ELISA kit (R&D Systems, Minneapolis, MN, United States). The absorbance was measured using a Spectra Max Microplate Reader (Molecular Devices, San Jose, CA, United States) with the associated software using the sandwich enzyme immunoassay technique.

### Histological Analysis

NP tissues from patients with CRS were obtained during surgery. Tissue was immediately fixed in 10% formalin, embedded in paraffin, and cut into thin sections. Sections were stained with hematoxylin–eosin. The number of eosinophils in the mucosa was counted per high power field (×400) in the three densest areas with a cellular infiltrate beneath the epithelial surface, and the mean number of eosinophils was calculated. Histological examinations were performed by three expert physicians unaware of the clinical data, as previously reported (Tokunaga et al., 2015).

### Cell Culture and Treatments

NP tissue was obtained from patients with CRS during nasal surgery. Human primary nasal fibroblasts were harvested from the tissues as previously reported (Yamada et al., 2019). Briefly, NP specimens were cut into pieces and then cultured in 10-cm dishes containing Roswell Park Memorial Institute (RPMI) 1640 medium (Nissui Pharmaceutical, Tokyo, Japan) supplemented with 10% heat-inactivated fetal calf serum (FCS) (Gibco, Grand Island, NY, United States), 0.29 mg/ml glutamine, 100 U/ml penicillin, and 100 μg/ml streptomycin, at 37°C, in 5% CO_2_ and humidified air. The NP specimens were removed after 24 h and the first passage was performed. Cells were harvested for 1–2 weeks until they reached confluency, and nasal mucosa-derived fibroblast cell lines were established. The purity of fibroblasts collected with this method was determined by immunohistochemical examination using cytokeratin and vimentin markers. We confirmed that the purity was higher than 98%. Fibroblasts were then seeded in 24-well plates for gene expression analysis and in 12-well plates for protein analysis. Once confluent, the submerged fibroblasts were stimulated with varying concentrations of recombinant leptin (Thermo Fisher Scientific), IL-4 (R&D Systems), and IL-13 (R&D Systems). Cells were then harvested at 24 h for real-time quantitative reverse transcription polymerase chain reaction (qRT-PCR) and 48 h for ELISA and Western blot analysis. After 24 h of stimulation, total RNA was isolated from the cells. For protein analysis, nasal fibroblasts were washed twice with 1 ml of ice-cold phosphate-buffered saline (PBS), and were then lysed with 0.15 ml of Mammalian Protein Extraction Reagent (M-PER; Thermo Fisher Scientific) supplemented with ProteoGuard EDTA-Free Protease Inhibitor Cocktail (Takara Bio, Shiga, Japan). Lysates were incubated on ice for 15 min and then clarified by centrifugation for 10 min at 14,000 × g, at 4°C. Supernatants were collected for ELISA and Western blot analysis.

### Real-Time PCR

Nasal tissues were immediately placed in a stabilization reagent (RNA later; (Thermo Fisher Scientific) and total RNA was extracted using NucleoSpin RNA II (Macherey-Nagel, Bethlehem, PA, United States) with DNase I (Thermo Fisher Scientific) according to the manufacturer’s instructions. The quality of total RNA from sinus tissue was assessed with a 2100 Bioanalyzer (Agilent Technologies, Santa Clara, CA, United States) using an RNA 6000 Nano LabChip (Agilent Technologies). Single-strand cDNA was synthesized using the High Capacity cDNA Reverse Transcription Kit (Thermo Fisher Scientific). Semiquantitative real-time RT-PCR was performed with a TaqMan method using an Applied Biosystems StepOnePlus Real Time PCR system (Thermo Fisher Scientific) in 15-μl reactions (7.5 μl of 2× TaqMan Master mix [Thermo Fisher Scientific], 0.75 μl of 20× primer and probe mixture). Probes for eotaxin-3 (Hs00171146_m1), the leptin receptor (Hs00174497_m1), and Glyceraldehyde 3-phosphate dehydrogenase (GAPDH) were purchased from Thermo Fisher Scientific. Aliquots of cDNA equivalent to 10 ng of total RNA were used for real-time PCR. The mRNA expression levels were normalized to the median expression of the housekeeping gene GAPDH.

### Leptin Receptor siRNA-Mediated Silencing

Fibroblasts established from NPs were plated in the wells of a 12-well plate (5 × 10^5^ cells/well). At 70% confluency, cells were then transfected with siRNA targeting human Leptin receptor (LEPRHSS106018; Thermo Fisher Scientific), lipofectamine 2000 (Thermo Fisher Scientific), and Opti-MEM (Thermo Fisher Scientific), according to the manufacturer’s instructions. Cells were harvested at 24 h for qRT-PCR and at 48 h for ELISA and Western blot analysis.

### Western Blot Analysis

The total protein concentration of the tissue homogenate from cultured nasal fibroblasts was measured using a Bicinchoninic acid Protein Assay Kit per the manufacturer’s instructions (Thermo Fisher Scientific), and 20 ng of protein was loaded for electrophoresis on a Mini-PROTEAN^®^ TGXTM Gel (Bio-Rad Laboratories, Hercules, CA, United States). The protein was then transblotted using a Trans-Blot^®^ TurboTM Transfer Pack and Trans-Blot^®^ TurboTM system (Bio-Rad Laboratories). Following transfer, the blots were blocked with 5% non-fat dry milk in 0.1% Tween 20/Tris-buffer saline (TBS) and TBS alone, and then incubated with anti-leptin receptor antibody (1:100; Novus Biologicals, Centennial, CO, United States). They were then incubated with a horse radish peroxidase-conjugated (HRP-conjugated) anti-rabbit IgG antibody (Dako; Agilent Technologies, Santa Clara, CA, United States). Subsequently, the blots were developed using chemiluminescent Western blot detection reagents (Amersham ECL Prime; GE Healthcare, Chicago, IL, United States) according to the manufacturer’s instructions. The blot density was scanned using Fusion Solo S (Vilber, Marne-la-Vallée cedex, France).

### Statistical Analysis

Differences between the groups were analyzed using the Kruskal-Wallis analysis of variance with Dunnett’s *post hoc* test, the Mann-Whitney U test, Chi-squared test, and Fisher’s exact test. The *q*-value based on the false discovery (FDR) was calculated to adjust for multiple comparisons using the Benjamini-Hochberg (BH) method. Correlations were assessed using Spearman’s rank correlation. In all cases, a value of *p* < 0.05 or a *q*-value were considered to be statistically significant. All statistical analyses were performed using GraphPad Prism 5.0 (GraphPad Software, La Jolla, CA, United States) software.

## Results

### Characteristics of the Patients

In total, 40 patients with ECRS, 15 patients with non-eosinophilic CRS (non-ECRS), and 12 without CRS (control) were included in the study. Blood samples from control subjects were obtained at the time of septoplasty, ethmoid cyst surgery, and submucosal turbinectomy. Table 1 shows the characteristics of the control, non-ECRS, and ECRS patients included in the study. There were no significant differences in age, total serum IgE, C-reactive protein (CRP), or body mass index (BMI) among the groups. Patients with ECRS showed significantly higher comorbidity with asthma compared to non-ECRS patients (*p* < 0.05). A higher proportion of eosinophils in peripheral blood was found in ECRS patients (*p* < 0.05 vs. control, *p <* 0.001 vs. non-ECRS). ECRS patients also displayed an elevated number of eosinophils in NPs and higher JESREC scores compared to those of non-ERS patients (*p* < 0.0001).

### Leptin Levels in Serum

**FIGURE 1:**
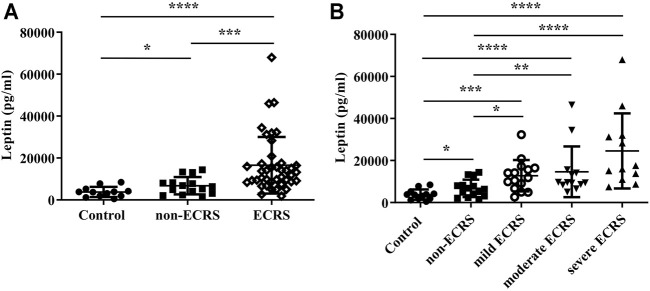
Levels of leptin in serum. **(A)** Serum leptin levels were higher in CRS patients than in control subjects (●: control; ■: non-ECRS; ◇: ECRS; *n* = 12–40). **(B)** Serum leptin levels were higher in patients ECRS subjects than in those with non-ECRS and control subjects (●: control; ■: non-ECRS; ○: mild ECRS; ▼: moderate ECRS; ▲: severe ECRS; *n* = 12–15). *p < 0.05; ***p < 0.001; ****p < 0.0001.

The serum leptin levels in both the ECRS and non-ECRS groups were significantly higher than those in control subjects (*p* < 0.0001, *q*-value < 0.0001 vs. ECRS; *p* < 0.05, *q*-value < 0.05 vs. non-ECRS) (Figure 1A). Serum leptin levels were also significantly higher in ECRS patients compared to non-ECRS patients (*p* < 0.001, *q*-value < 0.001) (Figure 1A). Furthermore, all ECRS subgroup patients showed elevated leptin levels compared to both non-ECRS patient subgroups (*p* < 0.05, *q*-value < 0.05 vs. mild ECRS; *p* < 0.001, *q*-value < 0.01 vs. moderate ECRS; *p* < 0.0001, *q*-value < 0.001 vs. severe ECRS) and control subjects (*p* < 0.001, *q*-value < 0.001 vs. mild ECRS; *p* < 0.0001, *q*-value < 0.0001 vs. moderate ECRS; *p* < 0.0001, *q*-value < 0.0001 vs. severe ECRS) (Figure 1B). We also found that serum leptin levels were significantly correlated with JESREC score (r = 0.3378, *p* < 0.05; Supplementary Figure S1), but not total IgE (data not shown) or BMI (Supplementary Figure S2).

### Relationship Between the Levels of Leptin and Eosinophils in Peripheral Blood and Eosinophils in NPs

**FIGURE 2:**
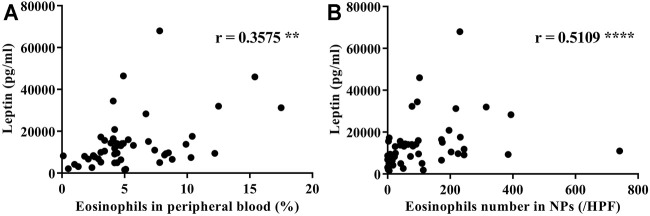
Correlations between serum leptin levels in CRS subjects and **(A)** the proportion of eosinophils in peripheral blood and **(B)** the number of eosinophils in nasal polyp tissue (*n* = 55). Correlations were assessed using a Spearman’s rank correlation test. **p < 0.01; ****p < 0.0001.

It is known that eosinophils take part in the pathogenesis of NPs (Schleimer, 2017; Takabayashi et al., 2019). Furthermore, in ECRS, both the number of infiltrated eosinophils in NPs as well as the proportion of eosinophils in peripheral blood are critical (Tokunaga et al., 2015). Importantly, eosinophils express the active isoform of leptin receptor (LEPR), and leptin enhances the survival and chemotaxis of eosinophils; these events are vital in the pathogenesis of Th2 inflammation (Conus et al., 2005; Wong et al., 2007; Kato et al., 2011; Yamamoto et al., 2013; Zheng et al., 2016; Zheng et al., 2018). Therefore, to test the potential involvement of leptin in CRS, we determined whether there is a relationship between the levels of leptin and the proportion of eosinophils in peripheral blood. We found a significant correlation between leptin and eosinophils in peripheral blood (r = 0.3575, *p <* 0.01; Figure 2A). Next, we determined whether there is a relationship between the levels of leptin and the number of eosinophils in NPs. We found that serum leptin levels were significantly correlated with the number of eosinophils in NP tissues (r = 0.5109, *p* < 0.0001; Figure 2B).

### Leptin Enhances Eotaxin-3 Gene Expression in Nasal Fibroblasts

**FIGURE 3:**
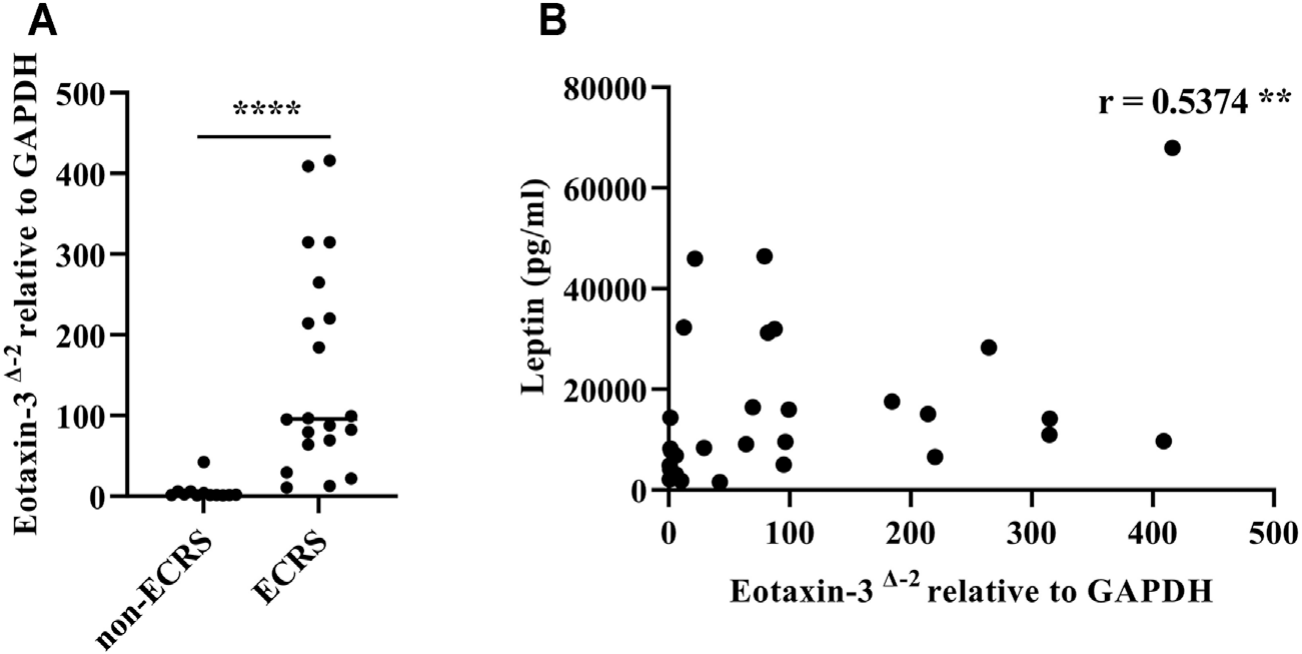
Eotaxin-3 gene expression in nasal polyp tissue. **(A)** Comparison of eotaxin-3 gene expression in nasal polyps and **(B)** correlation with leptin levels in serum. (*n* = 32). Correlations were assessed using Spearman’s rank correlation test. **p < 0.01; ****p < 0.0001.

**FIGURE 4:**
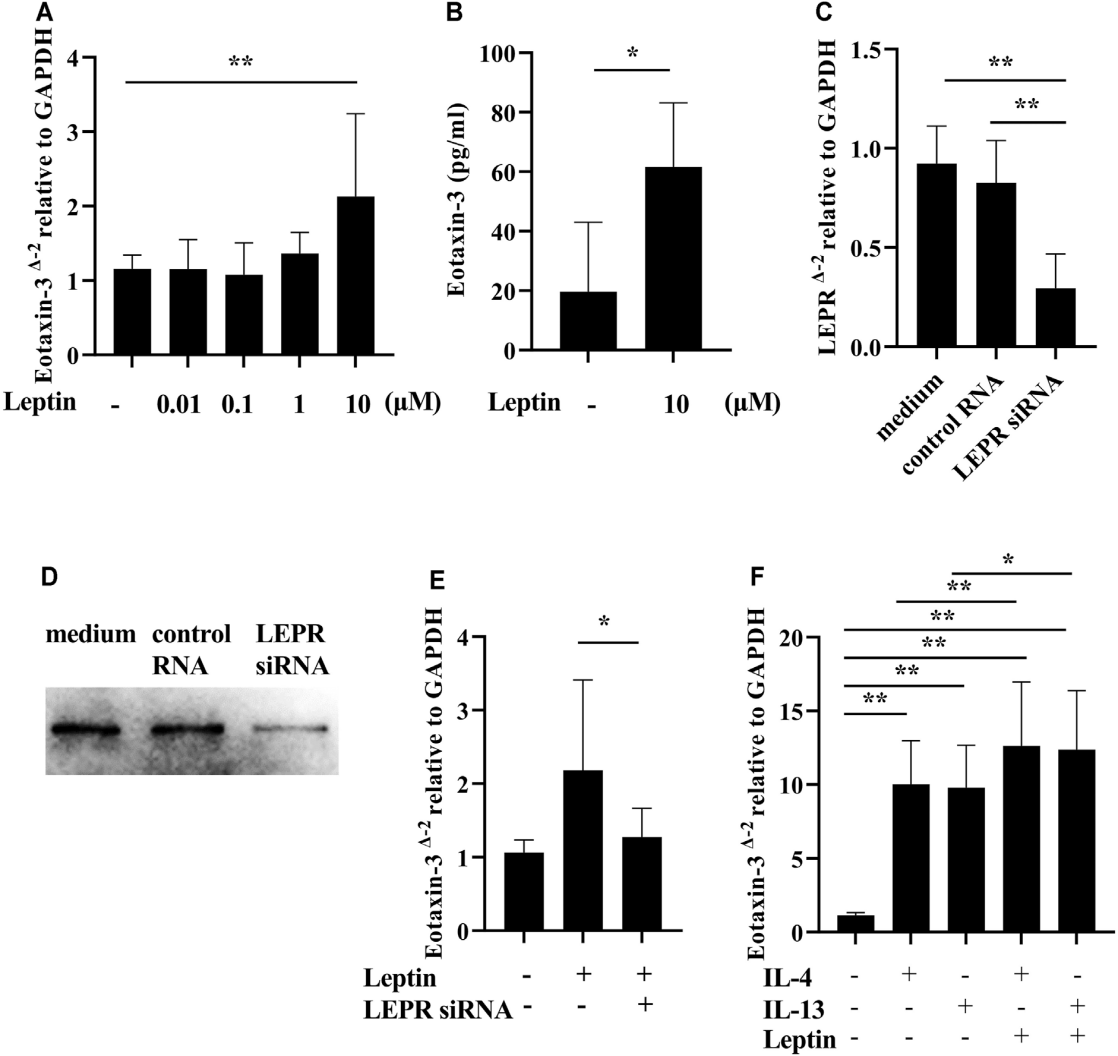
Induction of eotaxin-3 by leptin. **(A)** Submerged nasal fibroblasts established from nasal polyp cells were stimulated with varying concentrations of leptin (*n* = 8–10). **(B)** Submerged nasal fibroblasts were harvested after stimulation with 10 μM of leptin for 48 h, and whole cell lysates were collected. Levels of eotaxin-3 in the cell lysates and medium were measured by ELISA (*n* = 6). **(C**–**E)** Submerged fibroblasts were transfected with 500 nM of control RNA and siRNA targeting human leptin receptor. **(C)** Leptin receptor (LEPR) gene expression analysis. **(D)** Whole cell lysates were electrophoresed and transferred onto a PVDF membrane. A representative western blot with anti-human leptin receptor antibody is shown. **(E)** 48 h after transfection, cells were stimulated with 10 μM of leptin for 24 h (*n* = 8). **(F)** IL-4 (100 ng/ml) and IL-13 (100 ng/ml) with/without leptin (10 μM) for 24 h. Cell lysates were harvested for RNA to analyze eotaxin-3 mRNA expression by RT-PCR (*n* = 8). Data shown are mean ± SEM of four independent experiments. *p < 0.05, **p < 0.01 compared to non-stimulated cells.

It has been previously noted that eotaxin-3 (Chemokine (C-C motif) ligand 26: CCL26) immunoreactivity is elevated in NPs, and that eotaxin-3 protein levels are elevated in CRS (Stevens et al., 2015a; Yamada et al., 2019). Therefore, in order to evaluate the presence of eotaxin-3 in our surgical samples, we determined the levels of expression of eotaxin-3 mRNA in whole NP tissue extracts. Although the number of samples was limited (*n* = 32), the expression of eotaxin-3 mRNA in NPs from ECRS patients was significantly higher than that in NPs from non-ECRS patients (*p* < 0.0001, Figure 3A). Furthermore, there was a significant correlation between the levels of eotaxin-3 expression in NPs and the levels of leptin in the serum of the same patients (r = 0.5374, *p* < 0.01; Figure 3B). Given that previous reports have shown that nasal fibroblast from NPs express eotaxin-3 (Yamada et al., 2019), we stimulated nasal fibroblasts established from NPs with varying concentrations of recombinant leptin to determine whether leptin can enhance eotaxin-3 expression. The results showed that 10 μM of leptin significantly increased the expression of eotaxin-3 mRNA and protein in fibroblasts established from NPs (Figures 4A,B). In order to confirm the involvement of the leptin receptor in leptin-mediated induction of eotaxin-3, a small interfering RNA (siRNA)–knockdown approach was employed. Transfection with 500 nM of siRNA targeting the leptin receptor significantly suppressed the expression of leptin receptor mRNA and protein in fibroblasts established from NPs (*p* < 0.05, Figures 4C,D). The reduction in the leptin receptor gene was associated with a reduction of the induction of eotaxin-3 (Figure 4E). These results support the concept that enhancement of eotaxin-3 by leptin is dependent on the leptin receptor. Finally, we also stimulated NP derived fibroblasts with IL-4 and IL-13. As shown in Figure 4F, stimulation with either IL-4 or IL-13 enhanced eotaxin-3 expression. Furthermore, the combination of leptin and IL-4 or IL-13 further amplified eotaxin-3 expression (Figure 4F).

## Discussion

To the best of our knowledge, we are the first to report increased serum levels of leptin and to find that leptin levels are associated with disease severity in ECRS patients. As a prototypical adipocytokine, circulating leptin levels are known to reflect an individual’s amount of body fat, although leptin levels were not correlated with BMI in our study. Nevertheless, the findings of our study are in accordance with those of an important previous report indicating that elevation of leptin may be involved in the pathogenesis of asthma in a BMI-independent manner (Sood et al., 2006).

Notably, the JESREC study revealed the significance of elevations of eosinophils in both NP tissue (local) and in peripheral blood (systemic) in ECRS. According to the study, elevated proportion of peripheral eosinophils is one of the important clinical features in diagnosing ECRS. One of the most interesting findings of this study is that ECRS patients showed elevated leptin levels, and that both the proportions of eosinophils in peripheral blood as well as the number of eosinophils in NPs were closely correlated to the serum leptin levels. It has been previously reported that leptin prolongs cell survival for eosinophils and increases chemokinesis as well as the secretion of pro-inflammatory cytokines (Conus et al., 2005; Wong et al., 2007; Kato et al., 2011; Yamamoto et al., 2013; Zheng et al., 2016; Zheng et al., 2018). For example, Kato et al. revealed that leptin induces eosinophil chemotaxis and amplifies the chemotactic response to eotaxin (Kato et al., 2011). Given that chronic inflammation causes plasma leakage into tissue, the retention of several components in the plasma, including albumin, is one of the important features of eosinophil-driven inflammation in NPs (Bachert et al., 2000; Takabayashi et al., 2013; Imoto et al., 2019). The present results further demonstrate that eotaxin-3 gene expression levels are closely related to serum leptin levels and that leptin increases eotaxin-3 expression in nasal fibroblasts from NPs. Furthermore, the combination of IL-4 or IL-13 with leptin amplifies eotaxin-3 expression. Therefore, increased plasma leakage in NPs could potentially deliver circulating leptin to sinonasal tissue and affect eotaxin-3 expression in NPs.

It is well established that there are overlapping pathological features between asthma and CRSwNP. Although the patterns of inflammation are heterogeneous, both asthma and CRSwNP are regarded as primarily driven by type 2 mediated inflammation (Orlandi et al., 2016; Tan et al., 2017). In fact, type 2 inflammatory responses correlate with eosinophilia, which is generally associated with a higher prevalence and severity of asthma and CRSwNP (Lin et al., 2011; Wang et al., 2014; Stevens et al., 2015b; Schleimer, 2017). Furthermore, each disease effects the other, as asthmatic patients with CRS have an increased frequency of asthma exacerbations (Ek et al., 2013; Denlinger et al., 2017). Importantly, a recent report indicated that increased leptin levels are associated with the severity of asthma (Liang et al., 2016), which is in accordance with our findings that circulating leptin contributes to systemic eosinophilic airway inflammation in both asthma and ECRS.

Recently, emerging evidence has demonstrated the effectiveness of monoclonal antibodies (biologic therapy) targeting type 2 inflammatory markers. These biologic therapies provide promising results that support them as a potential treatment option for chronic airway diseases including CRSwNP (Bachert et al., 2019; Agache et al., 2020; Iqbal et al., 2020). In the treatment of severe eosinophilic asthma, elevated circulating blood eosinophils have been shown to be a predictive biomarker of the likelihood of better response to biologic therapies (Ortega et al., 2014; Chupp et al., 2017). Although the significance of monoclonal antibodies targeting type 2 cytokine has been shown, their effects may differ depending on the endotype of the individual patients’ disease (Iqbal et al., 2020). Along this line, we assessed the significance of circulating leptin in ECRS by focusing on the factors that are related to the recurrence of ECRS. We found that leptin levels are related to eosinophilia in both peripheral blood and NPs, and are also related to the severity of ECRS. Therefore, our findings suggest that it is worthwhile to further evaluate the role of leptin in ECRS, for example, by targeting leptin in a clinical trial.

The mechanisms underlying elevated leptin levels in ECRS patients are currently unknown. One possibility is that the amounts of adipokines and their physiological activities differ among different fat types and their distribution (Sood, 2011). Another possibility is that leptin levels are also influenced by food intake, such as a high fat diet (Chan et al., 2003; Murakami et al., 2007; Luglio et al., 2017). Notably, shifting toward a Western lifestyle with high caloric intake has been shown to cause leptin resistance (Dardeno et al., 2010; Heiss et al., 2021), which might further elevate leptin levels. Furthermore, central obesity, also known as abdominal obesity, may be another factor. To clarify these points, measuring total fat mass, especially in abdominal fat, might be helpful. Future studies that include detailed patient backgrounds are required, which may help shed light on the association between the recent increase in ECRS patients and the rapid change in urban lifestyles.

In conclusion, we found that the levels of leptin were elevated in ECRS patients compared to controls and non-ECRS patients. Furthermore, leptin levels were significantly correlated with the proportions of eosinophils in peripheral blood and the number of eosinophils in NPs. These results indicate that leptin may play an important role in the pathogenesis of ECRS in a BMI-independent manner.

## Data Availability

The original contributions presented in the study are included in the article/Supplementary Material, further inquiries can be directed to the corresponding author.
