# Supplementary material for: Elevated Serum Leptin Levels in Patients With Eosinophilic Chronic Rhinosinusitis
Source: Front Pharmacol. 2022 Jan 3;12:793607. doi: 10.3389/fphar.2021.793607 (PMC8762296; doi:10.3389/fphar.2021.793607)
Supplement: Supplementary file 1 [file DataSheet1.pdf]

Supplementary Material

1 Supplementary Figures

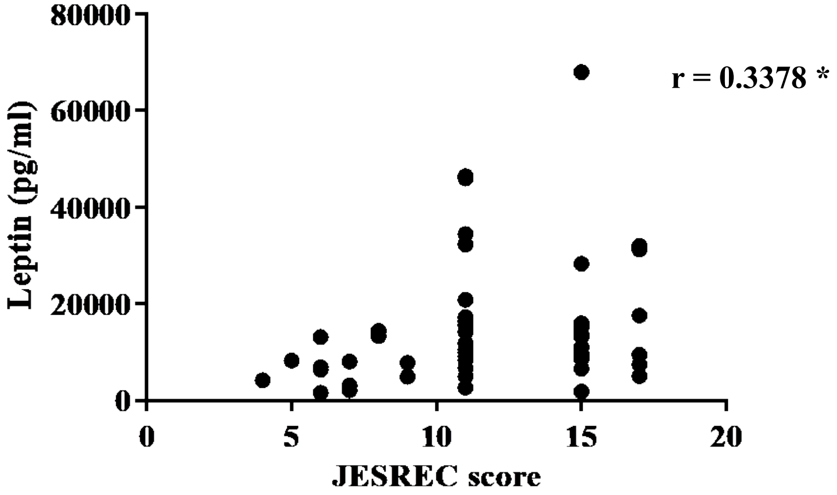

Supplementary Figure 1. Correlations between the serum leptin levels and JESREC score (n = 55). Correlations were assessed using Spearman's rank correlation test. JESREC, Japanese Epidemiological Survey of Refractory Eosinophilic Chronic Rhinosinusitis. *$P < 0.05$.

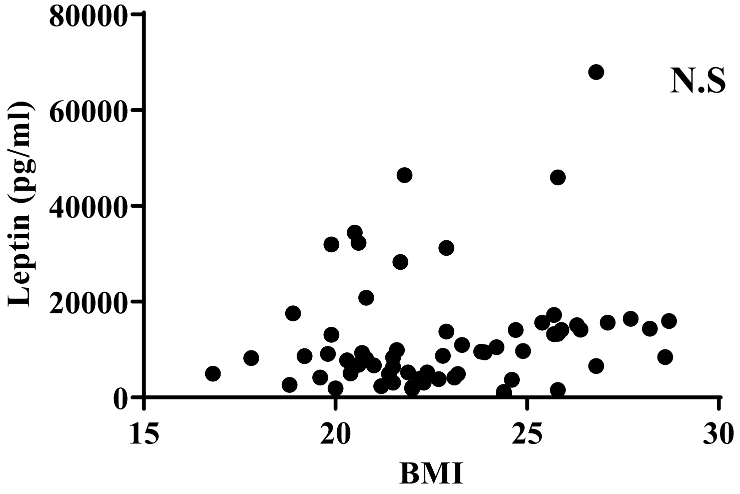

Supplementary Figure 2. Correlations between the serum levels of leptin and BMI (n = 67). Correlations were assessed using Spearman's rank correlation test. BMI, body mass index; NS, not significant.
